# Atrial Myxoma and Rheumatic Mitral Stenosis: Embolic Stroke Strikes Days Before Surgery

**DOI:** 10.7759/cureus.82666

**Published:** 2025-04-21

**Authors:** Tayyebah Jamal, Abdullah Hashmi, Hafsa Hashmi, Kashif A Hashmi, Ammar Akhtar, Muhammad S Khattak

**Affiliations:** 1 Cardiology, Nishtar Medical University, Multan, PAK; 2 Medicine, Quaid-e-Azam Medical College, Bahawalpur, PAK; 3 Medicine, Bakhtawar Amin Medical and Dental College, Multan, PAK; 4 Cardiology, Chaudhry Pervaiz Elahi Institute of Cardiology, Multan, PAK

**Keywords:** atrial myxoma, embolic stroke, myxoma complications, myxoma surgical resection, rheumatic mitral stenosis

## Abstract

Cardiac myxomas are the most common primary cardiac tumors. Their primary location is the left atrium, but they can arise in any cardiac chamber. Although histologically benign, atrial myxomas are functionally malignant due to their high embolic potential, which can lead to severe complications such as ischemic stroke. Left atrial myxoma can mimic mitral stenosis by causing obstruction to the blood flow at the level of the mitral valve. In developing countries, rheumatic mitral valve is the most common cause of mitral stenosis, and the concurrent occurrence of mitral stenosis and rheumatic heart disease is a rarity. We present a case of a 43-year-old man diagnosed with both conditions who developed an ischemic stroke due to tumor embolization two days prior to planned surgical intervention. This case underscores the importance of timely diagnosis and management in patients with concurrent cardiac pathologies.

## Introduction

Cardiac myxomas are the most common primary cardiac tumors, although primary cardiac neoplasms are very rare, with a reported prevalence of 0.03% in the general population and an age-adjusted incidence of 1.6 per million in the global population. Cardiac myxoma can arise in any chamber, but the most common site is the left atrium (75%) [1]. While they can remain asymptomatic, they often present with systemic symptoms, embolic events, or obstructive cardiac manifestations [2]. Embolic phenomenon due to myxomas in the left atrium occurs in 30%-40% of the patients, with the most common site being the central nervous system, leading to ischemic stroke [3]. Left atrial myxoma has a higher potential to embolize due to high pressure in the left ventricle during systole and motion of the mitral valve leaflets [4]. Myxoma itself contributes to embolization by shearing fragments into circulation. Left atrial myxoma can cause functional mitral stenosis by causing obstruction at the mitral valve [5]. In the developing world, rheumatic mitral valve is the major cause of mitral stenosis [6]. The coexistence of atrial myxoma and rheumatic mitral stenosis is very rare. This case illustrates the complex disease burden and the need for an individualized management approach.

## Case presentation

We describe a case of a 43-year-old man who presented to the medical emergency with a half-hour history of sudden-onset altered level of consciousness and weakness in his left arm and left leg. The altered level of consciousness was sudden in onset, accompanied by dizziness and 1-2 episodes of vomiting. It was associated with drooping of the angle of the mouth on the left side, with no observation of seizures. He had no history of smoking and other conventional risk factors such as diabetes, hypertension, and dyslipidemia. Two weeks prior to this presentation, he was admitted to the hospital with complaints of shortness of breath and cough on exertion. Workup revealed a left atrial myxoma, and the patient was allocated a surgical intervention time. However, two days prior to the planned surgery, he developed a stroke. On examination, the patient was confused and had a Glasgow Coma Scale (GCS) score of 12/15 (eye: 4, verbal: 3, motor: 5). He had a blood pressure of 100/70 mmHg, pulse rate of 98/minute, respiratory rate of 20/minute, and oxygen saturation of 98% on room air. Further neurological examination showed motor strength of 5/5 in the right upper as well as lower limbs, and 2/5 in the left upper and 3/5 in the left lower limb, along with a positive Babinski sign on the left foot. Sensory examination was not performed as the patient was not sufficiently alert. There was a visible drooping of the angle of the mouth on the left side, but the patient was able to close his eyes. A cardiovascular examination was carried out, revealing a loud S1 and an early diastolic low-pitched plopping sound, best appreciated at the apex with variance in position.

Investigations

CT Scan of the Brain

Non-contrast CT scan of the brain was done in the emergency department, which ruled out any hemorrhage. Because CT was done within an hour of the development of symptoms, it did not reveal any infarcted area. Given the history and examination findings, the lesion was localized to the right middle cerebral artery (MCA). MRI of the brain was planned to be done at a later stage.

Echocardiography

**Figure 1:**
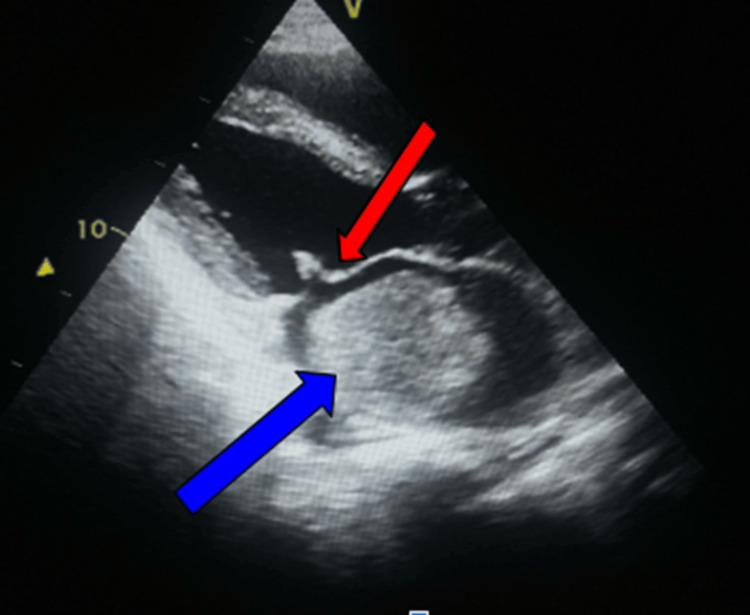
Transthoracic echocardiography (parasternal long-axis view) showing a large mass in the left atrium, consistent with an atrial myxoma (arrow) Thickened mitral valve leaflets and doming of the anterior mitral leaflet form a characteristic hockey stick appearance of the rheumatic mitral valve.

**Figure 2:**
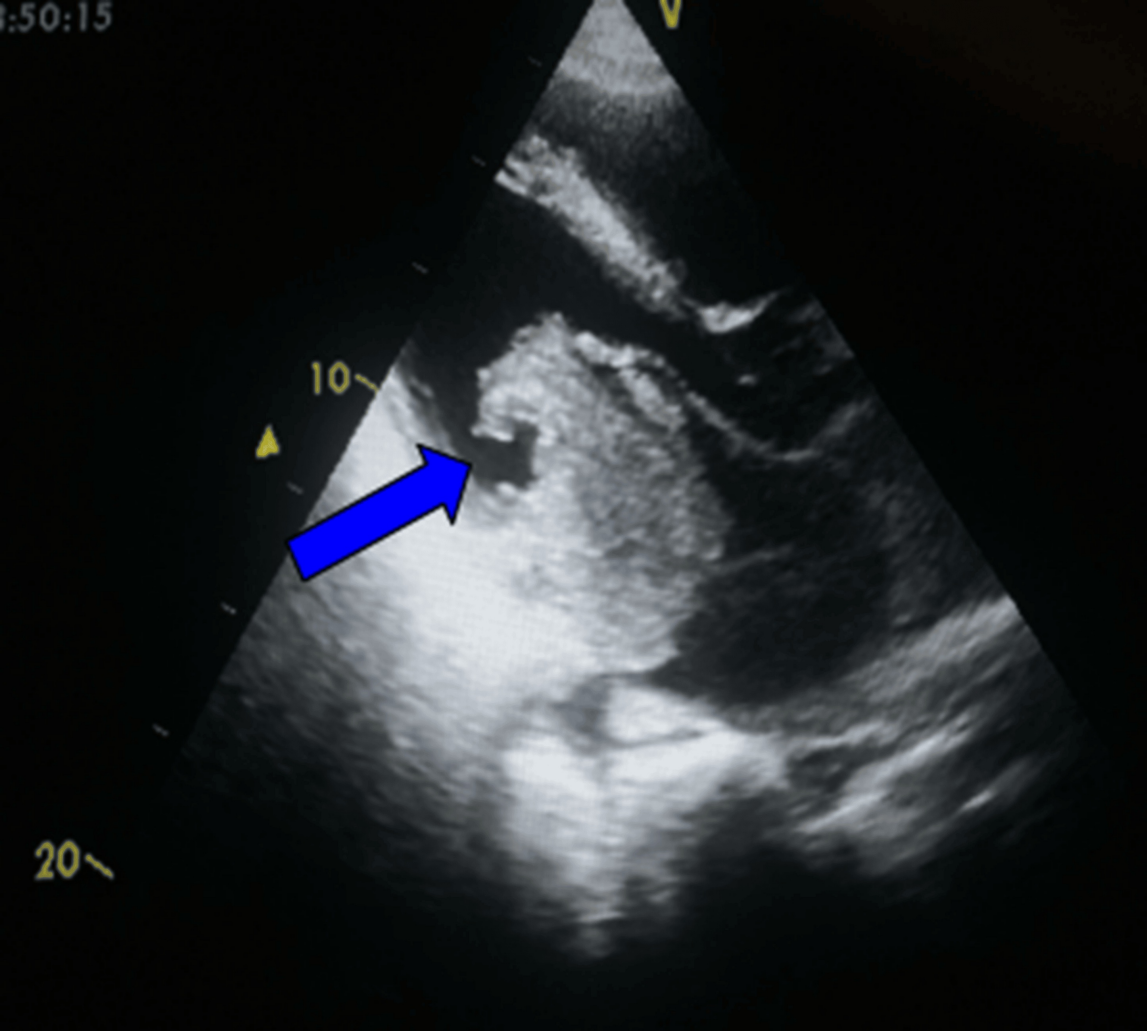
Transthoracic echocardiography (parasternal long-axis view) showing a mobile mass in the left atrium prolapsing through the mitral valve during diastole (arrow)

**Figure 3:**
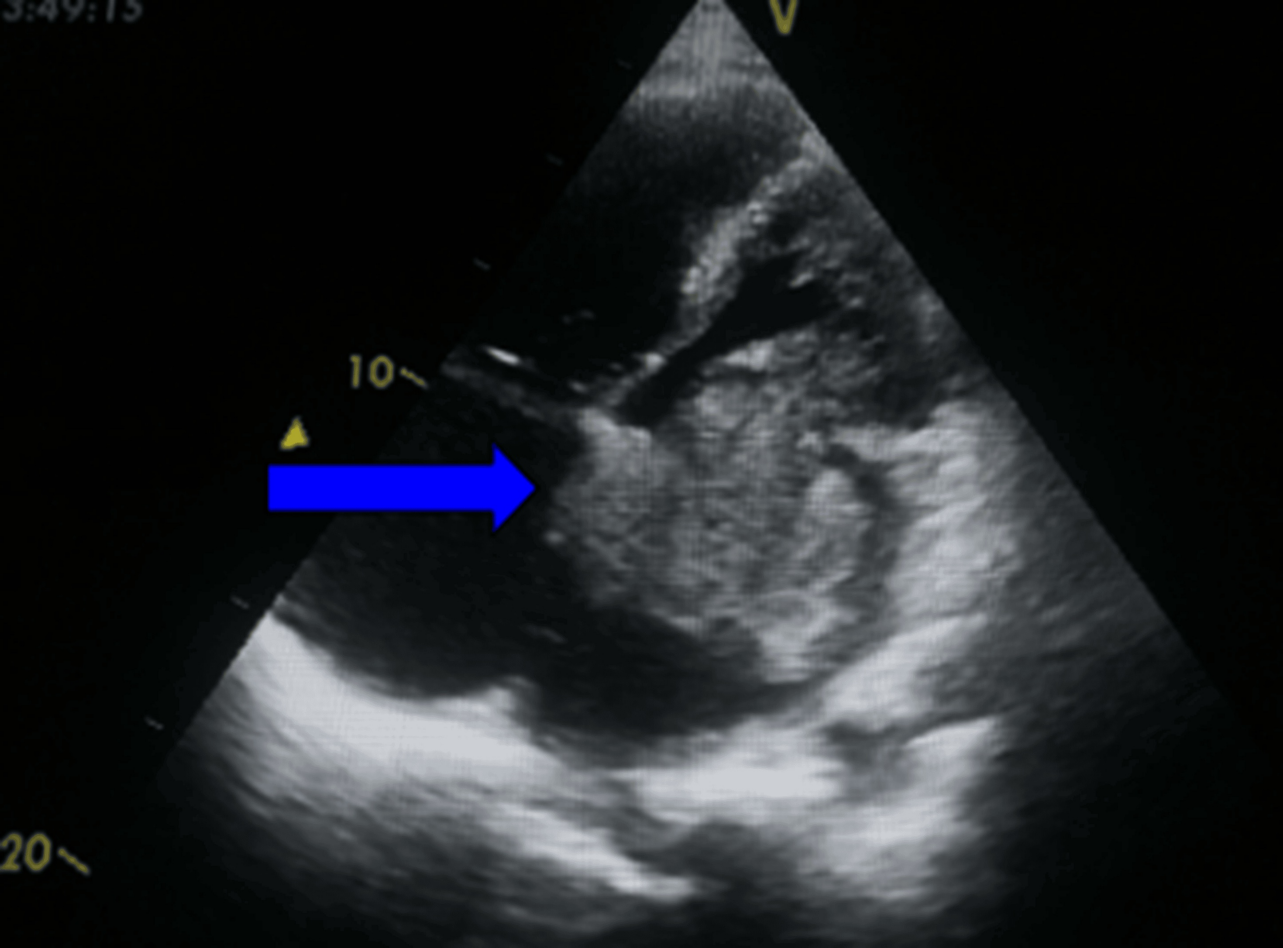
Transthoracic echocardiography (apical four-chamber view) showing a pedunculated mass attached to the interatrial septum that is obstructing the mitral valve during diastole (arrow)

Transthoracic echocardiography showed a large pedunculated mass in the left atrium, measuring 3.7 × 3.9 cm, attached to the interatrial septum and prolapsing through the mitral valve in diastole with associated rheumatic mitral stenosis (Figures 1-3). Findings included a rheumatic mitral valve with mild to moderate mitral stenosis. The mitral valve area was calculated by planimetry, which came out to be 1.8 cm^2^. Continuous wave Doppler study shows mitral valve pressure gradient (MVPG) (P) of 14 mmHg and MVPG (M) of 9 mmHg.

Management

**Figure 4:**
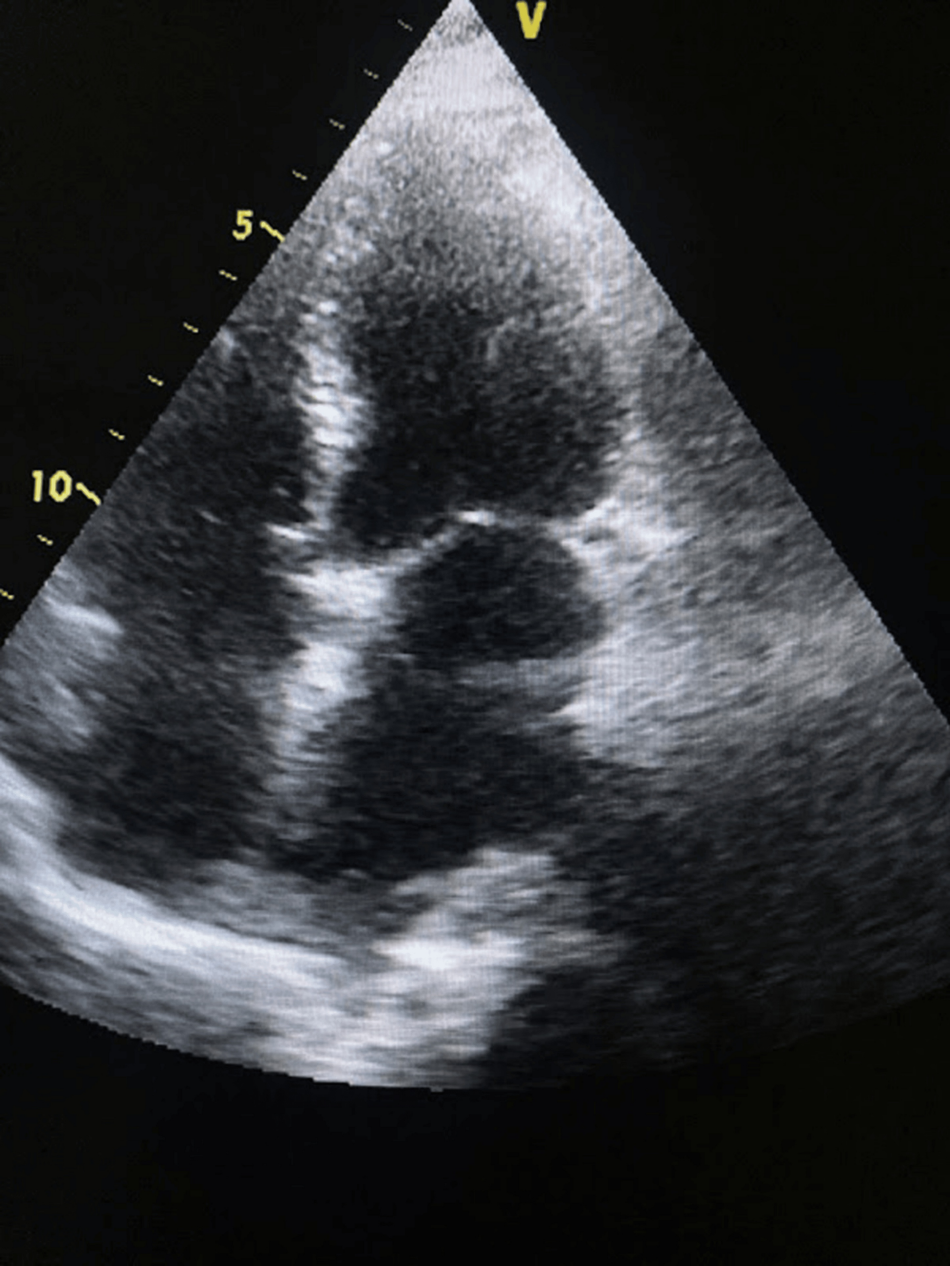
Transthoracic echocardiography (performed on the fourth postoperative day) (apical four-chamber view) showing no residual myxoma tissue

**Figure 5:**
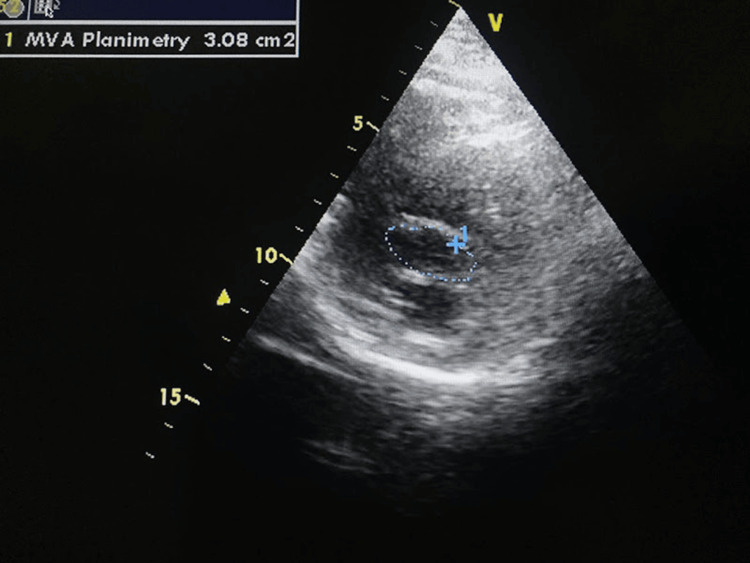
Transthoracic echocardiography (parasternal short-axis view) at the level of mitral valve leaflets demonstrating mitral orifice during diastole Manual tracing of mitral orifice shows a mitral valve area of 3.08 cm², which is within the normal range, indicating no residual mitral stenosis following open mitral commissurotomy.

Given the patient's deteriorating neurological and cardiac status, surgical intervention was deemed too high risk at this stage, as surgeons were reluctant to perform surgery on this patient due to the recent episode of stroke. However, after stabilization of the patient over the period of two weeks following the initial presentation with stroke, the patient underwent surgery, which included tumor resection and open mitral commissurotomy (Figures 4, 5).

## Discussion

Atrial myxomas are benign neoplasms derived from mesenchymal cells, usually present as a pedunculated mass attached at the fossa ovalis on the left side of the atrial septum, accounting for approximately half of cardiac tumors. The symptoms of atrial myxoma vary from dyspnea, cough, and heart failure-like symptoms, and ultimately can lead to stroke, making demise unfortunately a possibility [7].

Our patient had surgery planned, and two days prior to the surgery, he suffered an ischemic stroke, due to which the surgery was denied as it carried risks now. As far as ischemic stroke is concerned, only symptomatic treatment was undertaken. This case report highlights, as done by Chiarello et al., the optimum time for surgical removal of the tumor to mitigate further complications [8].

Sadia et al. presented a quite similar case in which a 44-year-old patient presented with right-sided weakness lasting for eight hours, except that the patient had already suffered a stroke, in contrast to our case. Upon investigation with echocardiography, similar to our case, it was found that the patient had an atrial myxoma blocking the mitral orifice. The patient underwent a non-contrast CT to find out the cause of hemiplegia, and a stroke was found, implicating the potential complication of myxoma. Similar to our case, surgery was opted after the onset of stroke; the patient had surgical removal of the tumor, preceded by treatment with unfractionated heparin [9].

Another case was presented by Shrestha et al., in which a 37-year-old woman presented with a brief loss of consciousness and weakness in the right side of her body. Similar to our case, while awaiting the planned surgery, the patient suffered another stroke, and the surgery was dismissed due to the cons in this instance. The patient was treated symptomatically and two weeks later died [10].

Anticoagulation therapy would be of benefit to reduce the incidence of thrombotic strokes in these patients, but again, it does not minimize the risk of tumor embolization as evidenced by the case series. Additionally, anticoagulation during a stroke attack could cause massive bleeding [11]. Surgical intervention is curative; however, surgical intervention during an acute ischemic attack is the point of debate. There is yet no consensus on the time frame between neurological stroke and cardiac surgical intervention [10].

Our case underlines the importance of a multifaceted approach regarding anticoagulation therapy after ruling out stroke and timely resection of cardiac myxoma. Like our patient, the pros and cons of early surgical intervention and late surgical intervention should be taken into account to prevent any probable unwanted events and maximize patient recovery.

## Conclusions

This case highlights the complex interplay between atrial myxoma and rheumatic mitral stenosis, emphasizing the need for timely intervention and a multidisciplinary approach. However, in high-risk cases where surgery is not immediately feasible, alternative management strategies must be considered. Nonetheless, it must also be kept in mind that surgery is the only definitive treatment.
